# Isolated Ulnar Neuropathy After Acute Angioedema

**DOI:** 10.7759/cureus.44872

**Published:** 2023-09-07

**Authors:** Sabine Itani, Hamza Malick, Priya C Nangrani, Tony Lai, Patrick Sims

**Affiliations:** 1 Medicine, Texas A&M College of Medicine, Bryan, USA; 2 Physical Medicine and Rehabilitation, Baylor University Medical Center, Dallas, USA

**Keywords:** neuropathic pain, ulnar neuritis, angioedema, electrodiagnostic, ulnar nerve

## Abstract

Ulnar neuropathy commonly causes hand paresthesia, often associated with mechanical compression or repetitive movements across the elbow or wrist. There are a few cases that document ulnar nerve injury from rapid compression in the absence of trauma. We present a 30-year-old previously healthy male who developed bilateral hand and forearm swelling, numbness, and pain after an allergic reaction initially treated with epinephrine and steroids. Following treatment, swelling improved; however, paresthesia and weakness persisted. Electrodiagnostic studies performed two months later showed severe ulnar neuropathy prominent at the left proximal wrist, confirmed by ulnar motor inching studies. Signs of acute or subacute denervation and active reinnervation were noted in the left flexor digitorum profundus and abductor digiti minimi. Right-sided studies were unrevealing, although magnetic resonance imaging (MRI) showed an acute flexor pollicis longus tear. Given the timing of events, it was felt that the ulnar neuropathy and acute muscle tear were related to the rapid onset of angioedema. Further research should be conducted on how acute episodes of angioedema (allergy) can cause nerve compression in different extremities. There are very scant reports of different types of angioedema (such as vibratory or hereditary) associated with neuropathy; however, there are no reports of acute allergic angioedema associated with neuropathy. A more comprehensive understanding of the pathophysiology of neuropathy following acute angioedema will help guide treatment approaches both acutely and after symptom presentation.

## Introduction

Ulnar neuropathy presents with hand paresthesia, numbness, and tingling. It is prevalent in 5% of the adult population, most commonly due to mechanical compression of the ulnar nerve at the elbow or wrist [1,2]. However, rare instances of angioedema causing ulnar neuropathy have been reported in the literature. We present a case of a patient with localized angioedema in the bilateral upper extremities that was treated with epinephrine and antihistamines but demonstrated persistent ulnar neuropathy after the resolution of allergic symptoms.

## Case presentation

A 30-year-old previously healthy male presented to urgent care with sudden onset of bilateral hand swelling and erythema. The patient reported a throbbing pain that worsened as the swelling continued to develop over the next 24 hours. The patient was febrile and tachycardic and endorsed an urticarial rash on both upper extremities with significant pruritus. The patient denied any prior history of allergies, allergic reactions, or relevant history of rheumatologic diseases and inflammatory arthropathy. He denied any exposure to animal or insect bites and no routine changes causing suspicion of contact dermatitis. He was diagnosed with angioedema and immediately treated with epinephrine, antihistamines, and a five-day course of steroids. Following the completion of the treatment course, the patient endorsed significant improvement in swelling, as well as a complete resolution of urticaria and erythema.

**Figure 1:**
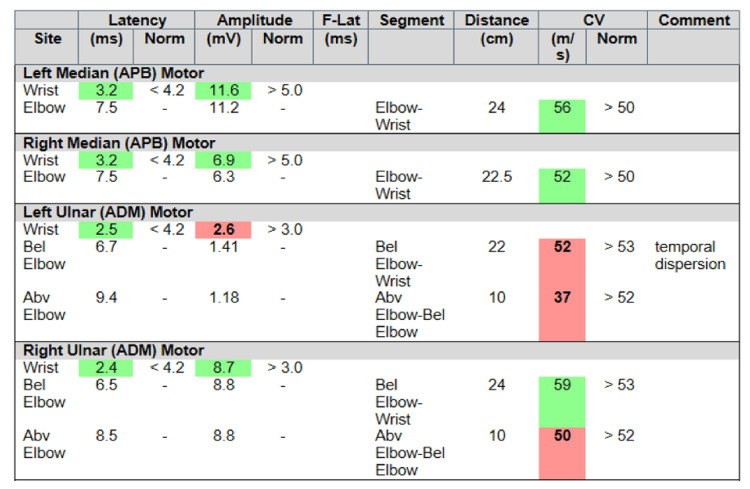
Motor nerve conduction findings significant for severe left motor ulnar nerve neuropathy APB: abductor pollicis brevis, ADM: abductor digiti minimi

**Figure 2:**
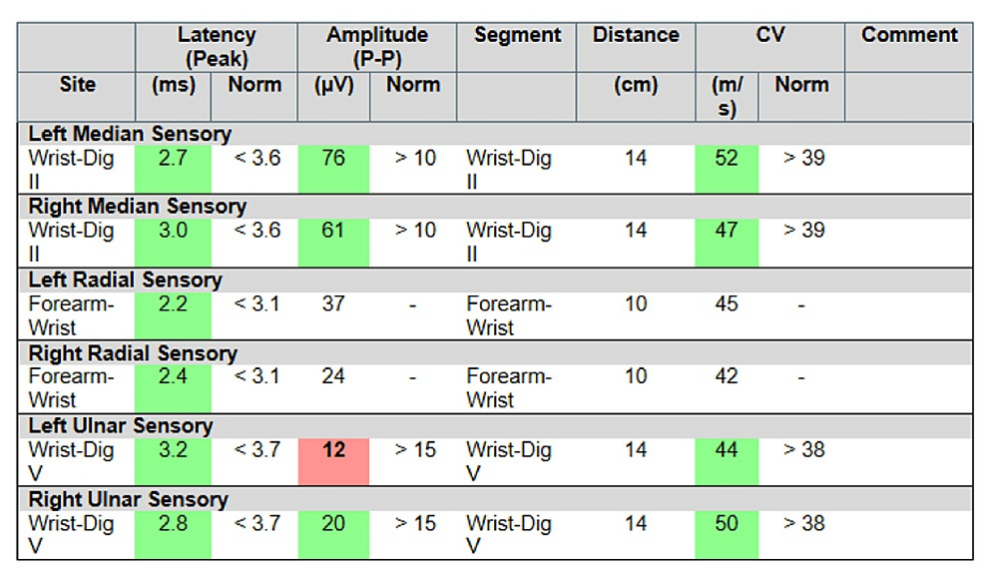
Sensory nerve conduction findings significant for severe left sensory ulnar nerve neuropathy

However, over the following three weeks, the patient developed worsening hand and wrist pain with associated numbness, tingling, and paresthesias in the fourth and fifth digits bilaterally. He was also noted to have ⅗ strength in flexion of the right thumb and left fifth digit. No stiffness, locking, muscle atrophy, or edema was noted. Physical examination was also notable for a positive Tinel test bilaterally at the wrist, a negative Tinel test bilaterally at the elbow, and a negative Phalen test. The results of routine laboratory studies, including urinalysis, complete blood counts, liver function tests, serum electrolytes, and C-reactive protein levels, were normal. The patient was treated with conservative management and rehabilitative hand exercises with a one-month follow-up for electrodiagnostic studies. Results of the electrodiagnostic studies performed revealed severe sensory and motor ulnar neuropathy prominent at the left proximal wrist (Figures 1, 2). Ulnar nerve function at the level of the elbow and proximal forearm was normal, further suggesting an etiology of distal ulnar nerve compression, and was confirmed by ulnar motor inching studies. Signs of acute denervation and active reinnervation were noted in the left flexor digitorum profundus and abductor digiti minimi. Ultrasound of bilateral wrists revealed thickening of the nerve. Moreover, magnetic resonance imaging (MRI) of the wrists revealed bilateral nerve enlargement. An incidental right-sided, acute flexor pollicis longus tear was discovered as well.

The patient continued to have constant pain and paresthesias in both hands over the following month; however, no recurrent episodes of swelling in the bilateral upper extremity were noted. He wore a wrist brace with mild improvement in pain symptoms and was referred to an orthopedic hand specialist for reconstruction of the right flexor pollicis longus.

## Discussion

Ulnar neuropathy is most commonly characterized as a form of compressive neuropathy occurring at the ulnar groove and hypothenar eminence. It is most commonly associated with repetitive movements and chronic external compression of the nerve [1]. Ulnar neuropathy presents with decreased grip strength and variable sensory involvement including numbness, tingling, and paresthesias, as well as localized tenderness with muscle atrophy in the fourth and fifth digits [1,2]. However, symptoms can often range from pure sensory deficits to pure motor deficits or a combination of both [2]. Instances of angioedema causing neuropathy have previously been reported in the literature. Spontaneous urticaria, idiopathic angioedema, and patients with hereditary angioedema have been reported to experience intermittent neuropathic pain secondary to nerve compression [3]. Vasculitis-associated neuropathy is also an exceedingly rare cause of angioedema in patients with underlying rheumatologic conditions and inflammatory dermatoses that exhibit vasculitic injury to peripheral nerves causing neuropathic pain [4]. However, an isolated case of a localized allergic reaction with persistent neuropathy secondary to acute angioedema is a novel entity. The pathophysiology behind this phenomenon is unknown; however, it may be caused by mast cell activation and the consequent TH2-driven eosinophilic inflammation, leading to changes in the behavior of afferent neurons, central nervous system neurons, and neurons in sympathetic and parasympathetic ganglia. These alterations lead to an immediate and pronounced stimulation of nerves, with long-lasting hyperexcitability [5].

The pathophysiology behind neuropathic pain secondary to this allergic angioedema may also be explained by the release of bradykinin, which activates primary sensory neurons and can trigger the release of neuropeptides such as substance P, neurokinin A, and calcitonin gene-related peptide. These neuropeptides play a vital role in neurogenic inflammation. Bradykinin, known for its potent vasodilatory effects via endothelial B2 receptors, can further exacerbate neurogenic inflammation by promoting processes such as plasma protein extravasation, alterations in endothelial cells, platelet aggregation, and the release of inflammatory neuropeptides and mediators, including serotonin [6]. Whether the cause of angioedema may be attributed to mast cell or bradykinin-mediated activation, the diagnosis of these entities remains a clinical diagnosis. A more comprehensive understanding of the pathophysiology behind these potentially different entities may help guide treatment approaches both acutely and after symptom presentation.

Similarly, diagnosis of ulnar neuropathy at the wrist is often accomplished through careful history and clinical examinations. In most cases, the diagnosis can be confirmed by electrodiagnostic testing or imaging. Physical examination should include an examination of the limb for muscle wasting, asymmetry, or deformity. Additionally, the ulnar nerve should be palpated along its course, and sensation to pinprick and light touch should be tested in all three major ulnar sensory areas [7]. When the clinical presentation is not straightforward, electrodiagnostic testing can be performed. At the wrist, electrodiagnostic localization of ulnar neuropathy will demonstrate motor and/or sensory nerve conduction slowing or blocking with stimulation at the wrist and no evidence of a more proximal block or slowing [8]. Imaging with both magnetic resonance imaging (MRI) and ultrasonography can aid in clinical diagnosis. In this case, the ultrasound revealed bilaterally thickening of the nerve and increased echogenicity, consistent with ulnar neuropathy. MRI also revealed bilateral nerve enlargement, consistent with neuropathy.

Treatment includes both conservative and surgical management. Conservative management includes physical therapy, exercise, and steroid injections. Surgical intervention can be explored if conservative treatment is ineffective. Nonsurgical intervention is based on the etiology of nerve compression. In the case presented, neuropathy from allergic angioedema was treated with non-steroidal anti-inflammatory drugs (NSAIDs), antihistamines, and epinephrine for acute symptomatic relief. Most cases of acute angioedema are similarly treated with an aggressive combination of the aforementioned drugs before tailoring subsequent treatment based on clinical history, as in the case of hereditary angioedema, or follow-up for allergic reactions as was seen in the case of our patient. Rarely, refractory cases may require surgical decompression to maximize immediate relief [9].

## Conclusions

The case of persistent neuropathy after allergic angioedema is exceedingly uncommon with this being one of the first few cases of ulnar neuropathy persisting after an allergic-like reaction. In this case, the electrodiagnostic studies, clinical history, and MRI were essential in ruling out other possible differentials for the neuropathic pain experienced by the patient. The acute onset of angioedema with paresthesias and numbness was initially treated with epinephrine and steroids to improve swelling and acute flares similar to typical management of angioedema in allergic reactions. Further research should be conducted on how acute episodes of angioedema can cause nerve compromise in different extremities. A more comprehensive understanding of the pathophysiology of post-angioedema neuropathy will help guide treatment approaches both acutely and after symptom presentation.
